# Data analytics approach for melt-pool geometries in metal additive manufacturing

**DOI:** 10.1080/14686996.2019.1671140

**Published:** 2019-09-25

**Authors:** Seulbi Lee, Jian Peng, Dongwon Shin, Yoon Suk Choi

**Affiliations:** a School of Materials Science and Engineering, Pusan National University, Busan, Korea; b Materials Science and Technology Division, Oak Ridge National Laboratory, Oak Ridge, TN, USA

**Keywords:** Powder bed fusion (PBF) process, melt-pool, single track, machine learning, correlation analysis, 106 Metallic materials, 404 Materials informatics / Genomics

## Abstract

Modern data analytics was employed to understand and predict physics-based melt-pool formation by fabricating Ni alloy single tracks using powder bed fusion. An extensive database of melt-pool geometries was created, including processing parameters and material characteristics as input features. Correlation analysis provided insight for relationships between process parameters and melt-pools, and enabled the development of meaningful machine learning models via the use of highly correlated features. We successfully demonstrated that data analytics facilitates understanding of the inherent physics and reliable prediction of melt-pool geometries. This approach can serve as a basis for the melt-pool control and process optimization.

## Graphical abstract

**Figure:**
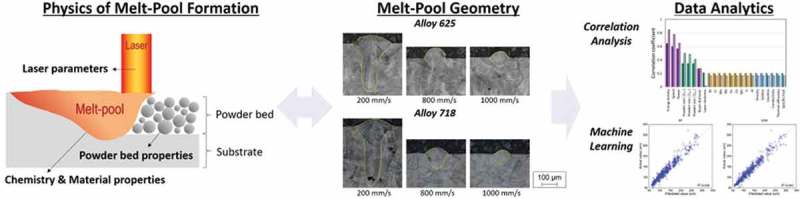

## 1. Introduction

Additive manufacturing (AM) offers many key benefits, which could change the industrial paradigm in various fields, as a tool-free, cost-efficient and digital approach to manufacturing [1]. Among the various metal AM processes, powder bed fusion (PBF) offers advantages for the fabrication of fully dense, near-net-shape metallic parts directly using high-energy heat sources, such as a laser and an electron beam. The PBF process comprises a sequence of layer depositions by selectively melting a powder layer (together with the previously deposited layer (or a substrate)), which leads to the formation of a series of melt-pools on a micrometer spatial scale, formed by harsh solidification conditions on a millisecond temporal scale. Here, increasing the reliability and stability of additively manufactured products as a function of processing conditions is a grand challenge.

The melt-pool geometry is important as a primary criterion for optimizing processing conditions because it is an indirect evidence of interactions between processing parameters and intrinsic materials properties. Also, there is a need to understand geometry-related phenomena, such as defect formation, microstructure evolution, thermo-mechanical properties, and so on. Extensive research, as well as computational analysis, has been performed to clarify the effects of materials and process parameters on melt-pool characteristics, and the underlying physics [2–7]. However, there are more than 130 processing parameters associated with the PBF process [8,9], which interact intricately with various phase changes over a wide temperature range during the process. It is difficult to understand the overall relationships among the processing variables through well-controlled experiments or simulations using only a few variables. Also, it is challenging to quantitatively determine which features have utmost priorities, and how relevant they are to the characteristics of AM-processed parts.

Herein, we introduce an emerging data analytics approach to predict the multi-physics-based phenomena in AM, along with a scientific insight into the underlying mechanisms [10–15]. To predict melt-pool geometries, machine learning training was performed using a database of PBF-processed single tracks of Alloy 625 and Alloy 718 powders. Through the correlation analysis, the machine learning models were improved using highly correlated features, which enabled us to interpret the underlying physical mechanisms. Finally, the results demonstrated that the reasonably trained data analytics approach prioritizes key materials/process parameters of the melt-pool formation, with the physical relevance, and facilitates the accurate prediction of melt-pool geometries for the AM process optimization.

## 2. Experimental and data analytics details

### 2.1. Data acquisition through systematic microstructural analysis

Pre-alloyed spherical powders of Alloy 625 (21.4Cr-9.0Mo-3.3Nb-3.6Fe-0.2Co–0.3Mn-0.3Ti-0.2Al-Ni, by Chang Sung Co.) and of Alloy 718 (19.2Cr-3.1Mo-5.0Nb-18.4Fe-0.2Co-0.05Mn-1.0Ti-0.5Al-Ni, by Carpenter Inc.), were used to fabricate single tracks on a substrate of the same material. The PBF machines used in this study were Mlab and M2 cusing (Concept laser) with maximum laser powers of 100 W and 400 W, respectively. The laser power, scan speed, beam diameter and layer thickness were used as process parameters for the PBF operation. Table 1 summarizes the laser parameters and powder bed properties under a wide range of process windows. All processes were performed at room temperature in an inert argon atmosphere.

**Table 1.** List of PBF process parameters for fabricating single tracks of alloy 625 and alloy 718 powders.

Processing parameter	Material (machine)		
	Alloy 625 (M2)	Alloy 718 (Mlab)	Alloy 718 (M2)
Power (W)	120, 180, 240	70, 80, 90, 100	120, 180, 240
Scan speed (mm/s)	200, 400, 600, 800, 1000	500, 700, 800, 900, 1100	200, 400, 600, 800, 1000
Beam diameter (μm)	50, 100, 150	50	50, 100, 150
Layer thickness (μm)	25, 50	25, 35, 45	25, 50
Powder size distribution – D_10_, D_50_, D_90_ (μm)	18, 31, 49	23, 34, 45	15, 30, 47
Total no. of the data set	175	117	180

**Figure 1.**
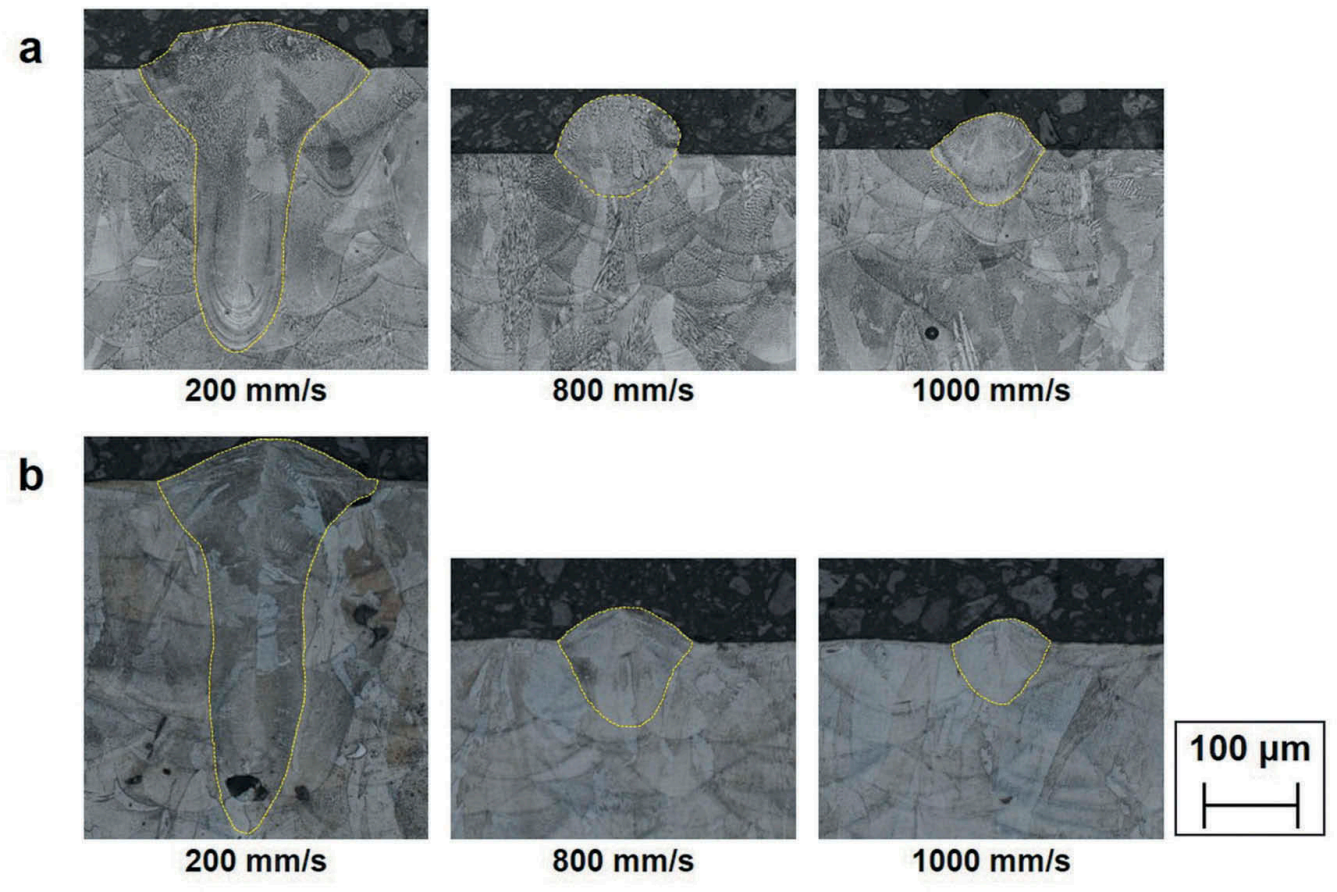
Metallographs of melt-pool cross sections for (a) Alloy 625 and (b) Alloy 718 as a function of the scan speed. Here, the laser power, beam diameter and layer thickness are 180 W, 50 μm and 25 μm, respectively.

**Figure 2.**
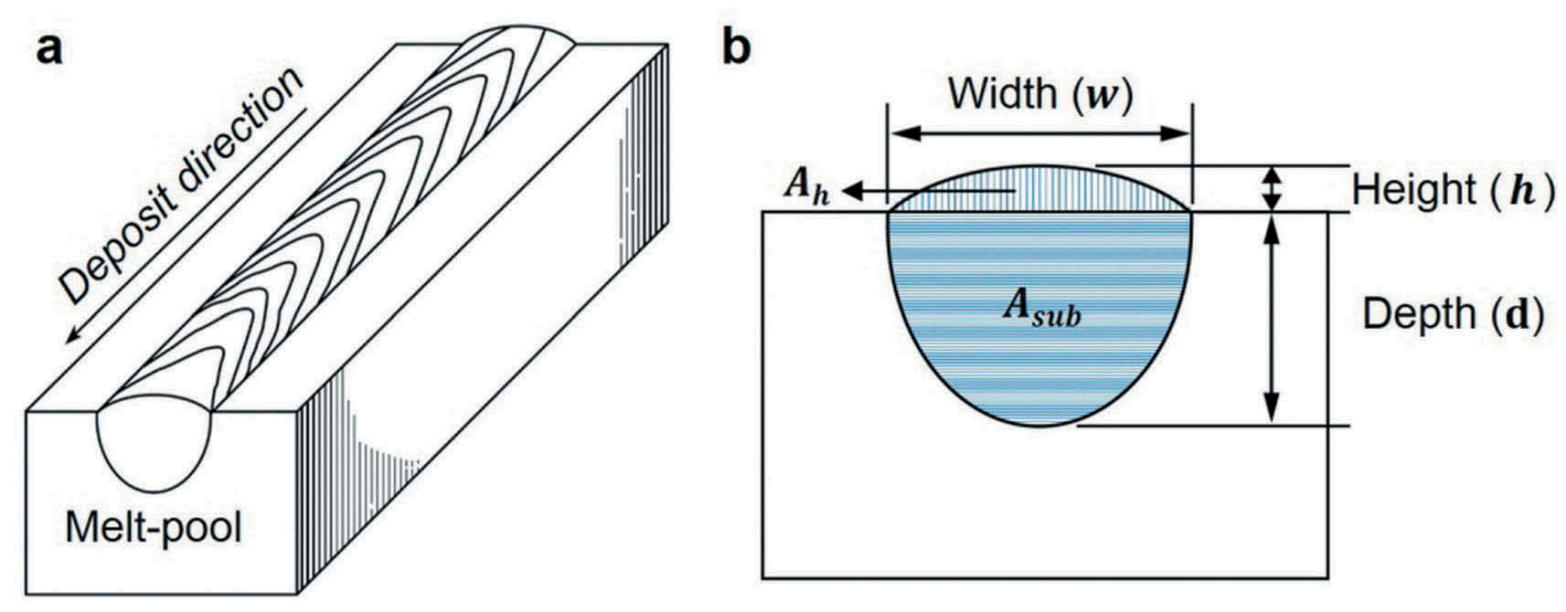
Schematic illustration of (a) single track deposition by the PBF process and (b) the melt-pool geometry parameters (targets) from the cross-section view.

For reliable data acquisition, sections in the middle of the 3-mm long single tracks were prepared for the metallography. Figure 1 presents the representative optical metallographs in a cross-section view, showing the melting mode from conduction to keyhole. As shown in Figure 2, the target output parameters (will be referred to as targets, hereafter) were set up such that they represent characteristics of melt-pool geometries: those measured in the substrate (width (*w*), depth (*d*), and area within the substrate (*A_sub_*) in Figure 2) and in the powder bed (height (*h*) and area based on the height (*A_h_*) in Figure 2). By measuring these target values through image analysis, a geometry database of 472 melt-pools was constructed (Table 1).

### 2.2. Data analytics

1. Correlation analysis

A total of 5 targets and 23 input features categorized by process parameters and material-related properties are listed in Table 2.

**Table 2.** List of input features and targets used in the present study.

Classification		Constituents
Input features	Chemistry of powders	Ni, Cr, Mo, Nb, Fe, Co, Mn, Ti, Al
	Materials thermal property	Solidus, liquidus, density, conductivity, thermal diffusivity, specific heat
	Information of the powder bed	Powder size distribution (D_10_, D_50_, D_90_), layer thickness
	Laser parameters	Power, scan speed, energy density*, beam diameter
Targets for the melt-pool geometry	Measured in the substrate	Width, depth, area within the substrate
	Measured in the powder bed	Height, area based on the height

* Energy density is given by $E_{\rho }=\frac{P}{v\;l}$ (herein, $E_{\rho }$ is the surface energy density, P is the power, v is the scan speed, and l is the layer thickness of powder bed.)

The correlation analysis between input features and targets was performed using both the advanced maximal information coefficient (MIC) approach and the conventional Pearson’s correlation coefficient (PCC) approach. The PCC approach is suitable for seeking the linear relationship between input features and target, while MIC approach can identify non-linear relationships of high-dimensional large dataset [10,15–17]. By using two different approaches, we expect to have insights for different statistical aspects between input features and target properties and then to inspire domain expert for understanding the physical phenomena [15]. In the PCC approach, coefficient value has between −1 and +1, where a positive value indicates a direct relationship and a negative correlation coefficient indicates a reciprocal relationship between input features and targets. The MIC belongs to the maximal information-based nonparametric exploration (MINE) [17], having between 0 to 1 coefficient value. To compare the results of both approaches, MIC values and absolute PCC values were used. Based on the quantitative comparison of these values, we can identify which physical phenomenon is predominant for each melt-pool formation and develop meaningful machine learning models using highly ranked features.

(2) Machine learning

In this study, six machine learning algorithms were employed for generality (i.e. not limited to a specific data analytic algorithm): Bayesian ridge regression (BR) [18], kernel ridge regression (KR) [19], linear regression (LR) [20], nearest neighbors regression (NN) [21], random forest regression (RF) [22], and support vector machine (SVM) [23]. All of these are in the open source data analytic toolkit ASCENDS [24]. Based on a 5-fold cross-validation, the predictability of machine learning models was evaluated by calculating the coefficient of determination (R^2^) between the actual and predicted values as a function of the number of top-ranked features (5, 10, 15, 20 and all) associated with each correlation approach.

## 3. Results and discussion

**Figure 3.**
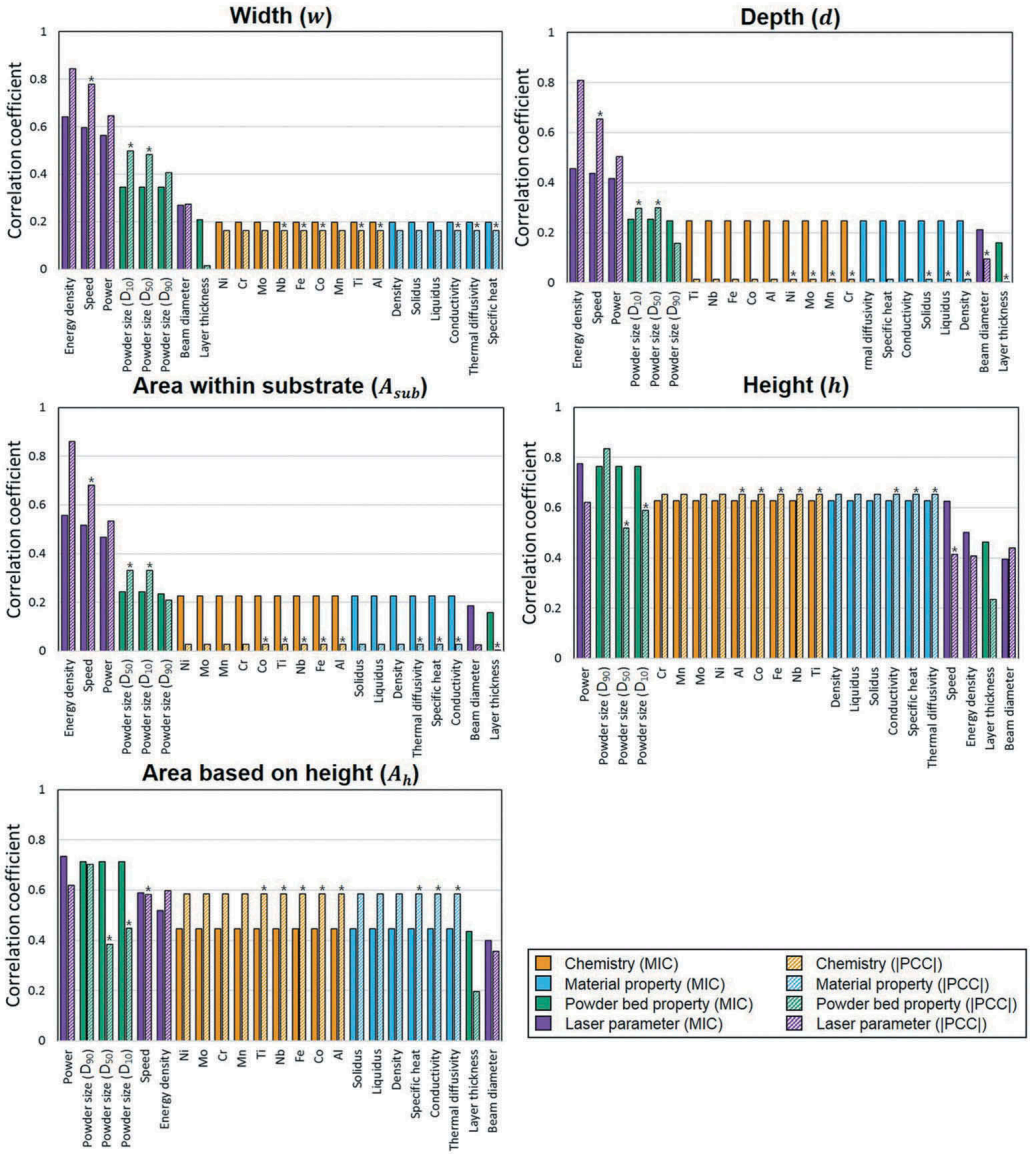
Results of correlation analysis using the MIC approach, compared with the absolute PCC values (bars marked by patterns), for the five targets (*w, d, A_sub_, h* and *A_h_*). In the plots, asterisk marks on the PCC bars indicate negative values.

Figure 3 shows the results of the correlation analysis for the five targets using both MIC and PCC approaches. Here, asterisk marks on PCC correlation bars indicate negative correlation coefficients, which mean inverse influence. The rank order of the features shows a distinct difference between the melt-pool geometries associated with the substrate (*w, d* and *A_sub_*) and with the powder bed (*h* and *A_h_*). Also, the rank orders are different depending on the correlation approach and targets, as listed in supplementary Table 1.

In Figure 3, the energy density, laser power, scan speed, and powder size distribution were highly ranked for most targets. Here, first three parameters are associated with how much energy is emitted by the heat source. The powder size distribution (in the powder bed) is related to the energy absorptivity. The laser beam interacts with the powder bed first, and multiple scattering between the powders causes a higher laser absorptivity than on the bare substrate. In particular, the laser absorptivity tends to be chiefly dependent on the powder size distribution in the top layer, which affected the primary reflection of the laser [25].

**Figure 4.**
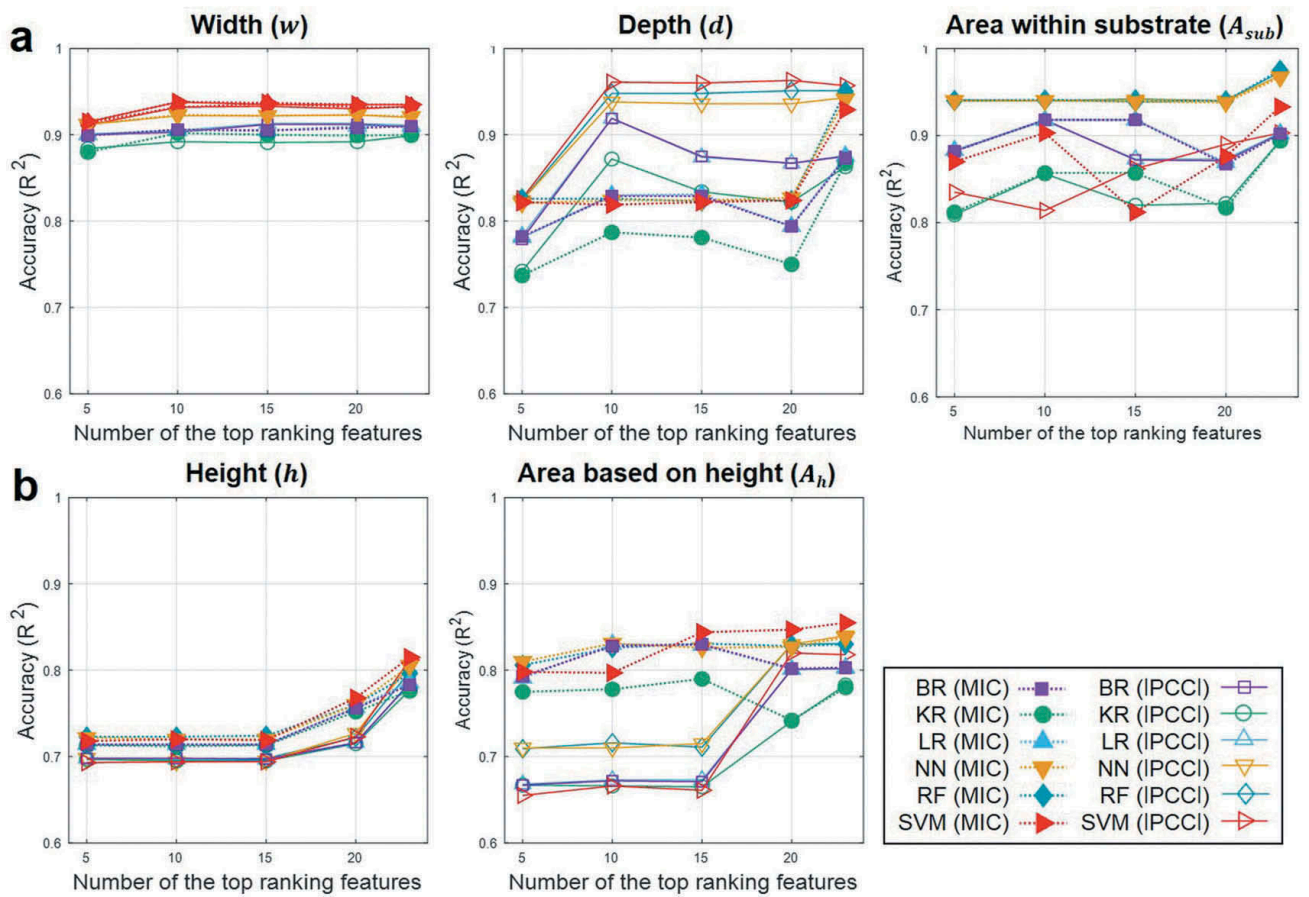
Accuracy analysis of the five targets (*w, d, A_sub_, h* and *A_h_*) as a function of the number of the top-ranked features (5, 10, 15, 20 and all), determined by the two correlation approaches (MIC and PCC), for the six different machine learning algorithms (BR, KR, LR, NN, RF and SVM). Here, R^2^-values are averages after 5 machine learning training cycles.

To identify the dependence for selected features based on correlation analysis, we conducted the accuracy analysis of 5 machine learning training cycles, as shown in Figure 4 (numeric values with standard deviations are listed in supplementary Table2).

In Figure 4, the accuracies (R^2^-values) show higher predictability for the melt-pool geometries associated with the substrate (*w, d* and *A_sub_*), when the PCC approach is employed, and higher predictability for the melt-pool geometries associated with the powder bed (*h* and *A_h_*) under the MIC approach (although *w* and *A_sub_* are highly predictable using both approaches). In general, the PCC approach is suitable for capturing linear relationships between the input features and targets; whereas the MIC approach is suitable for capturing a wide range of relationships, including linear, exponential, or periodic, or even all functional relationships [17]. A possible interpretation of the results is that melt-pool geometries measured in the substrate have linear relationships with the most highly ranked features, whereas those measured in the powder bed have complex relationships with the most highly ranked features. Also, the equitability of the MIC approach seems to facilitate interpretation of the relationships with noises [17] caused by surface defects, vaporization losses, height roughness, and so on. In the following, the main discussion will focus on correlation approaches with better machine learning accuracies (PCC for *w, d* and *A_sub_*, and MIC for *h* and *A_h_*).

In Figure 3, the energy density, laser power, scan speed, and powder size distribution ranked high for *w, d* and *A_sub_* based on the PCC approach. Chemistries and material properties followed in the rankings; they show different PCC correlation values between *w* (about 0.17), and *d* and *A_sub_* (≤ 0.03). Here, the similar correlation tendency for *d* and *A_sub_* is probably due to a proportionality between two targets. Considering the melt-pool formation under the laser irradiation, *d* is determined by direct laser penetration (the laser cavity) resulting from the largest recoil pressure. However, *w* is decided by the heat transfer, mainly through the convection of liquid flow surrounding the laser cavity [2,3,7].

This phenomenon was observed by Zhao et al [2], who conducted in situ observations of the melt-pool formation through the synchrotron X-ray imaging technique. The experimental evidence shows that the depth of the laser cavity is similar to the *d* value of the melt-pool, while the *w* value of the melt-pool continuously increases because the cavity has a constant width (similar to the beam diameter) during melt-pool evolution [2]. Herein, it can be seen that the PCC correlation value of the beam diameter for *w* is higher than that for *d*, also having a positive value (Figure 3). The computational study by Khairallah et al. [3] showed that the liquid metal under an incident laser moves away from the bottom to the rear part of the melt-pool through a dynamical vortex fluid for heat dissipation. In brief, the direct laser penetration is a major phenomenon that determines *d*, and the heat transfer through the fluid convection is a major physical phenomenon that determines *w*. The correlation analysis performed in the present study accurately captured such a difference.

It is interesting in Figure 3 that the powder size distribution, chemistries, and material properties have much higher correlation values for *h* and *A_h_* than do the targets associated with the substrate (*w, d* and *A_sub_*) for both PCC and MIC approaches. Since the melt volume above the substrate is determined by the amount of consolidated powders, the powder size distribution is the highly correlated feature for *h* and *A_h_* in terms of the powder packing density [26]. After the laser passes, the final *h* and *A_h_* values will be decided by the fluid characteristics during cooling. Since fluid characteristics are intrinsically based on material properties and chemistries, these features have high correlation values with melt-pool geometries associated with powder bed.

**Figure 5.**
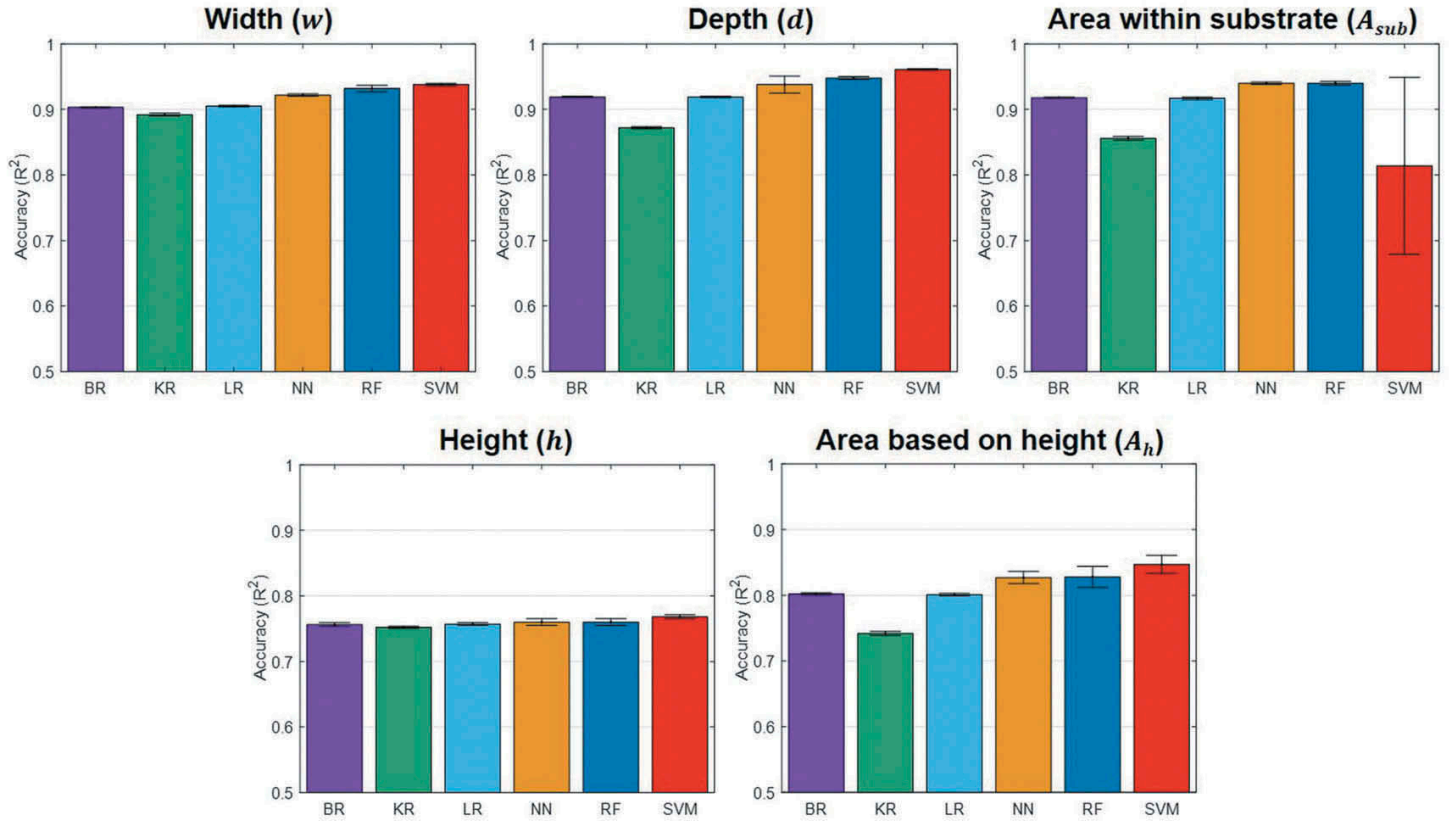
Accuracy analysis of the five targets (*w, d, A_sub_, h* and *A_h_*) for the six optimized machine learning algorithms (BR, KR, LR, NN, RF and SVM). Here, average R^2^-values and standard deviations are plotted after 5 machine learning training cycles.

Based on the accuracies of the trained models in Figure 4, the minimum numbers of top-ranked features, excluding noise-inducing features and including physically key features [15], were determined to be 10 and 20 for melt-pool geometries measured in the substrate (*w, d* and *A_sub_*) and the powder bed (*h* and *A_h_*), respectively. Figure 5 shows the resulting accuracies (R^2^-values) of the six optimized machine learning models.

In Figure 5, for *w, d* and *A_sub_*, the machine learning models show fairly high predictability, having a highest R^2^-value of more than 0.9, which is striking, considering a limited number of melt-pool data available for training. However, for *h* and *A_h_*, the results show rather low predictability, having R^2^-values between 0.75 and 0.85. The reason for the relatively low predictability of *h* and *A_h_* seems to be associated with the complexity of dynamic powder motions. During the laser irradiation, vapor-driven flow causes powder spatter ejection backward, upward, or even forward with respect to the scan direction [3–5]. In particular, the development of dynamic vapor flow as a result of uneven temperature distribution in the melt-pool makes it even more difficult to predict powder motion [2–5]. The powder entrainment toward the melt-pool caused by the interaction between the metal vapor and the surrounding gas pressure causes more complex powder motion [2,4,5]. Also, the difficulty of controlling the layer thickness uniformity over the powder bed seems to give an additional effect on the uncertainty of the powder bed properties [27]. Therefore, for the improvement of predictability for *h* and *A_h_*, it is suggested to incorporate input features representing the gas- and vapor-driven powder behaviors, and related to the material properties associated with optics and rheological characteristics. Also, we will consider the integrated input features (such as D_90_/D_10_ for the powder distribution, new energy density formula, and so on) and the new representative targets for defining the melt-pool geometries having physically meaningful insights.

## 4. Conclusions

Modern data analytics approaches were employed to check the feasibility of predicting melt-pool geometries of PBF single-tracks and the physical relevance to the real phenomena, and the following conclusions were drawn.

1. Machine learning models better predicted melt-pool geometries measured in the substrate with the PCC approach, and those measured in the powder bed with the MIC approach. It implies different interactions of input features and output targets by the laser irradiation to the powder bed and substrate.
2. The correlation analysis suggested that the depth, width and height of the melt-pool are physically relevant with the laser penetration, fluid convection, and fluid properties of melted powders, respectively.
3. Machine learning models optimized with high ranking features accurately predicted melt-pool geometries measured in the substrate, but showed relatively low predictability for those measured in the powder, which seemed to be associated with complex and dynamic powder motions.
4. Input features prioritized by the correlation analysis and machine learning training well captured the relevant physical phenomena to form melt-pools. A strong possibility of using modern data analytics to accelerate the PBF process optimization for the melt-pool control was demonstrated.

## Supplemental material

The supplementary data for this article can be accessed here.
