# Supplementary material for: Data analytics approach for melt-pool geometries in metal additive manufacturing
Source: Sci Technol Adv Mater. 2019 Sep 25;20(1):972–8. doi: 10.1080/14686996.2019.1671140 (PMC6818108; doi:10.1080/14686996.2019.1671140)
Supplement: Supplemental Material [file TSTA_A_1671140_SM7755.pdf]

Table 1. List of PBF process parameters for fabricating single tracks of Alloy 625 and Alloy 718 powders

Processing parameter	Material (machine)		
	Alloy 625 (M2)	Alloy 718 (Mlab)	Alloy 718 (M2)
Power (W)	120, 180, 240	70, 80, 90, 100	120, 180, 240
Scan speed (mm/s)	200, 400, 600, 800, 1000	500, 700, 800, 900, 1100	200, 400, 600, 800, 1000
Beam diameter (μm)	50, 100, 150	50	50, 100, 150
Layer thickness (μm)	25, 50	25, 35, 45	25, 50
Powder size distribution —D_{10}, D_{50}, D_{90} (μm)	18, 31, 49	23, 34, 45	15, 30, 47
Total no. of the data set	175	117	180

Table 2. List of input features and targets used in the present study

Classification		Constituents
Input features	Chemistry of powders	Ni, Cr, Mo, Nb, Fe, Co, Mn, Ti, Al
	Materials thermal property	Solidus, liquidus, density, conductivity, thermal diffusivity, specific heat
	Information of the powder bed	Powder size distribution (D_{10}, D_{50}, D_{90}), layer thickness
	Laser parameters	Power, scan speed, energy density, beam diameter
Targets for the melt-pool geometry	Measured in the substrate	Width, depth, area within the substrate
	Measured in the powder bed	Height, area based on the height
